# Pseudoglucagonoma Syndrome Following Frey’s Surgery: A Case Report of a Rare Presentation

**DOI:** 10.7759/cureus.58076

**Published:** 2024-04-11

**Authors:** Sai Kavya D, Sukesh Gautam S, Afthab Jameela Wahab

**Affiliations:** 1 Department of Dermatology, Saveetha Medical College and Hospitals, Saveetha Institute of Medical and Technical Sciences (SIMATS) Saveetha University, Chennai, IND

**Keywords:** enteroglucagon, frey's procedure, improved nutrition, pseudoglucagonoma syndrome, necrolytic migratory erythema

## Abstract

Pseudoglucagonoma syndrome is defined as the presence of necrolytic migratory erythema in the absence of a glucagon-secreting tumor. Necrolytic migratory erythema is the hallmark of glucagonoma syndrome but can also occur due to pancreatitis, pancreatic insufficiency, gastrointestinal dysfunction, inflammatory bowel disease, celiac disease, malabsorption disorders, nutritional deficiencies, hepatocellular dysfunction, and hypoalbuminemia. Pseudoglucagonoma syndrome is extremely rare, and the diagnosis is often delayed, resulting in delayed treatment.

We report a rare case of pseudoglucagonoma syndrome in a malnourished male patient following Frey’s surgery. The patient presented with a skin rash which gradually progressed over 20 days with diffuse hair loss. On cutaneous examination, multiple irregular erythematous and eroded plaques surrounded by a hyperpigmented scaly border were present over the dorsal aspect of the lower limbs, upper limbs, gluteal region, and genitals. Routine investigations showed normocytic normochromic anemia, neutropenia, lymphocytosis, dyslipidemia, and hypoalbuminemia. Rapid resolution of the skin lesions was observed with improved nutrition.

## Introduction

Glucagonoma syndrome is a rare, paraneoplastic phenomenon caused by a glucagon-secreting tumor of the alpha cells of the pancreas. It is associated with glucagon hypersecretion, which promotes glycogenolysis, gluconeogenesis, ketogenesis, and lipodieresis, resulting in elevated blood glucose levels and consequent development of diabetes mellitus. The clinical manifestations of glucagonoma syndrome include necrolytic migratory erythema (NME), weight loss, glossitis, cheilitis, steatorrhea, diarrhea, venous thrombosis, anemia, hyperglucagonemia, and neuropsychiatric disturbances. NME is the hallmark of this syndrome.

Pseudoglucagonoma syndrome is extremely rare and is defined as the presence of NME in the absence of a glucagon-secreting tumor. Pseudoglucagonoma syndrome can occur secondary to pancreatitis, malabsorption disorders, nutritional deficiencies, hepatocellular dysfunction, and hypoalbuminemia. Hence, these patients need frequent nutrition assessment, supplementation, and monitoring. As a delayed diagnosis of pseudoglucagonoma is a major issue, early diagnosis is necessary for better therapeutic outcomes and also to prevent complications. Here, we discuss a case of pseudoglucagonoma in a malnourished male patient secondary to chronic calcific pancreatitis for which Frey’s procedure was done two years ago.

## Case presentation

A 26-year-old male with type 1 diabetes mellitus presented with complaints of rash, pain, swelling, itching, and peeling of the skin over the lower limbs for 20 days. The rash gradually progressed to involve the upper limbs, groin, perianal, and genital region. There was a history of diffuse hair loss, loss of weight, and loss of appetite. The patient had a known case of chronic calcific pancreatitis for which Frey’s procedure - local resection of the pancreatic head combined with lateral pancreatojejunostomy - was done two years back.

**Figure 1:**
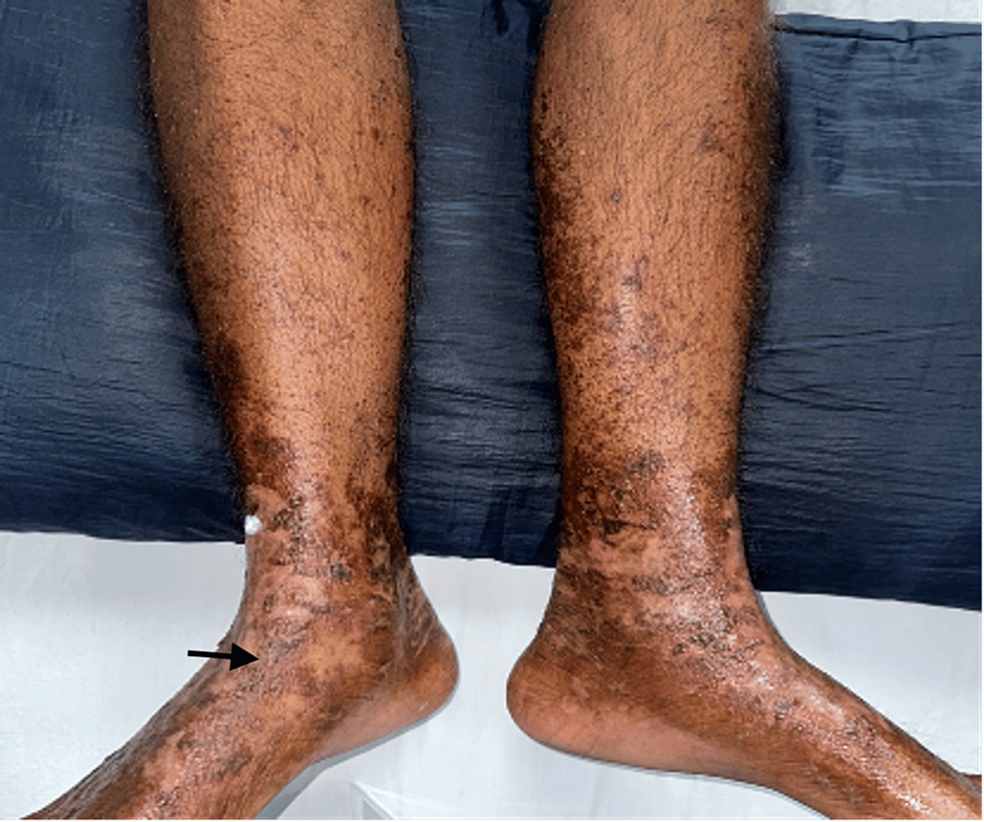
Multiple irregular erythematous and eroded plaques surrounded by a hyperpigmented scaly border.

**Figure 2:**
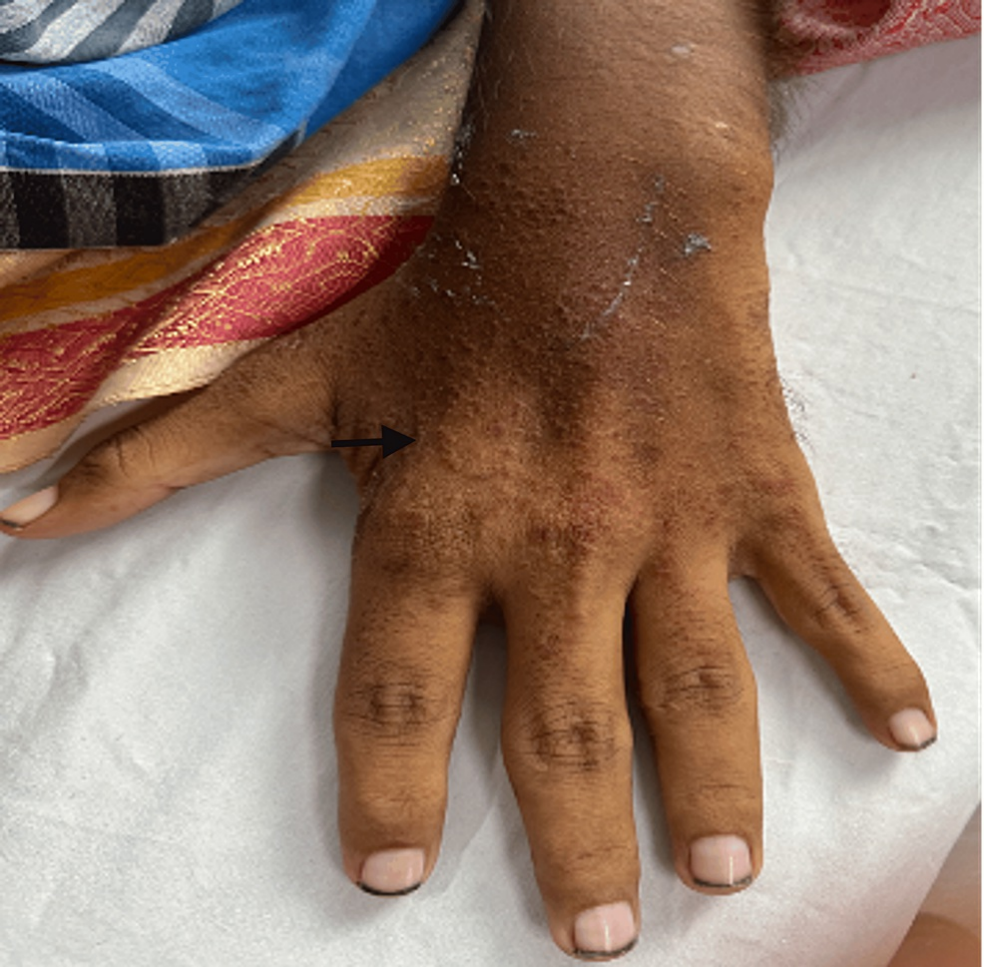
Multiple erythematous papules over the upper limb.

**Figure 3:**
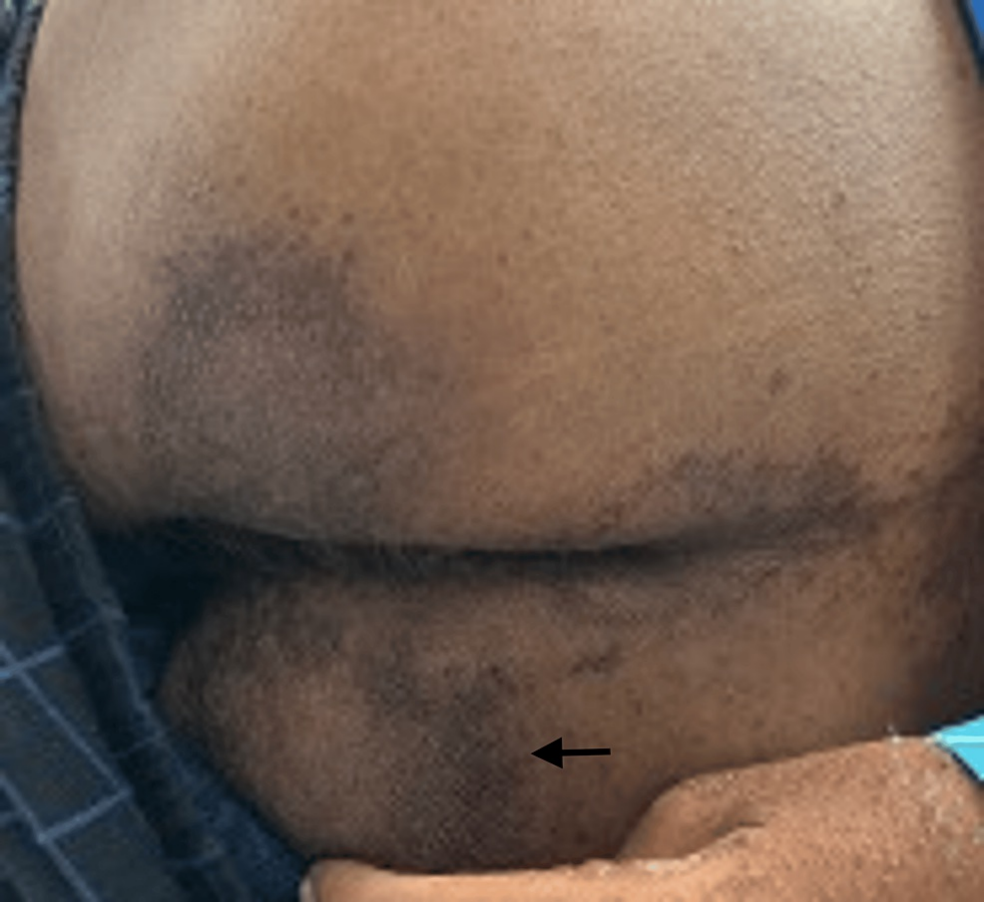
Hyperpigmented scaly plaques over the gluteal region.

**Figure 4:**
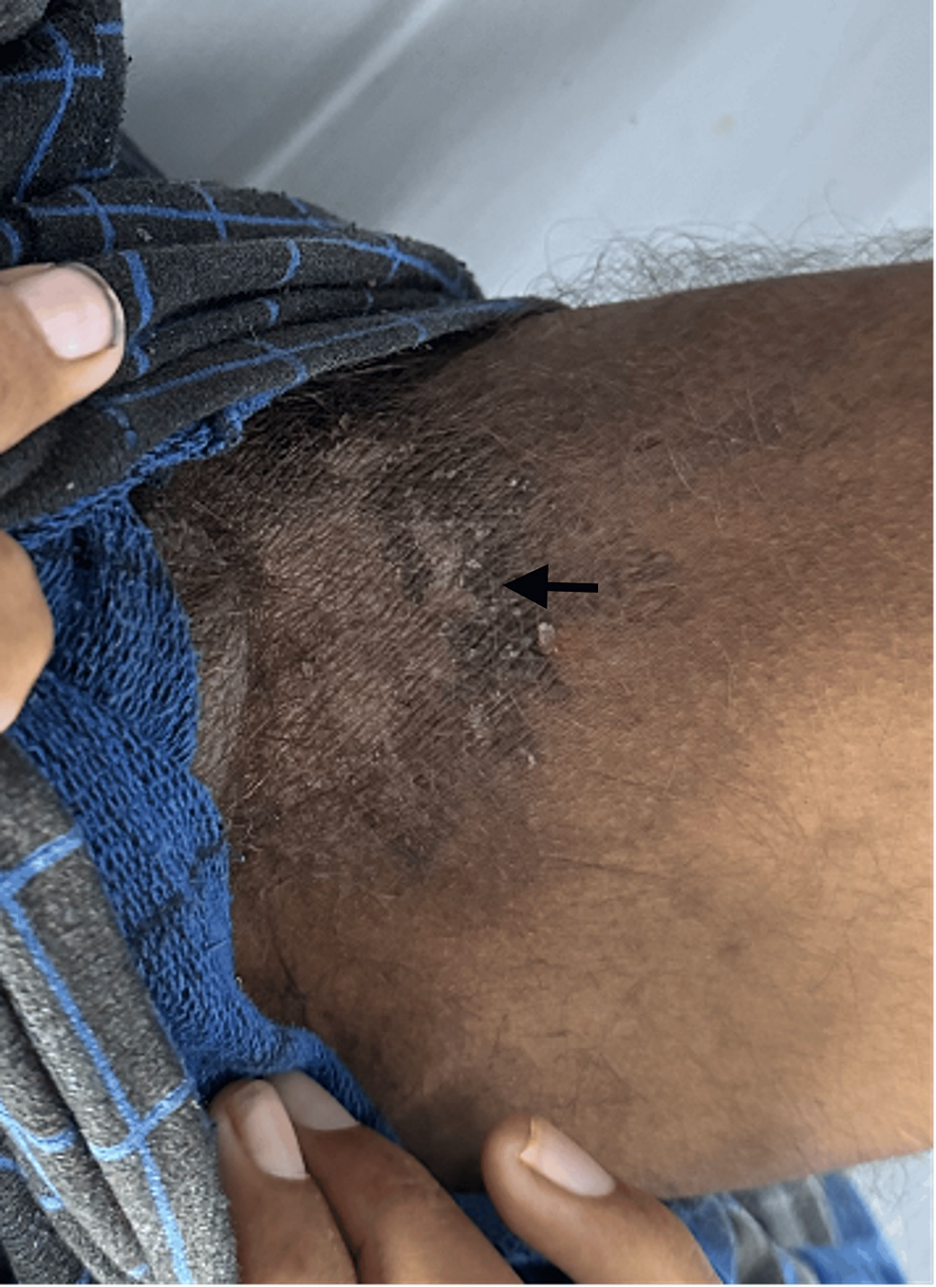
Hyperpigmented scaly plaques over the genitals.

On general examination, the patient was found to be thin-built and malnourished; vital signs and other systems were normal. On cutaneous examination, multiple irregular erythematous and eroded plaques surrounded by a hyperpigmented scaly border were present over the dorsal aspect of the lower limbs, upper limbs, gluteal region, and genitals (Figures 1-4, respectively).

On examination of the oral cavity, angular cheilitis was present. A hair examination revealed light-colored scalp hairs with diffuse non-cicatricial alopecia.

Routine investigations showed normocytic normochromic anemia, neutropenia, lymphocytosis, dyslipidemia, and hypoalbuminemia. Erythrocyte sedimentation rate, blood sugar, renal function test, liver function test, pancreatic function test, serum electrolytes, and serum glucagon were within normal limits. Chest X-ray and ultrasonogram (USG) of the abdomen were normal. Individual vitamin and mineral levels could not be done as the patient could not afford it.

**Figure 5:**
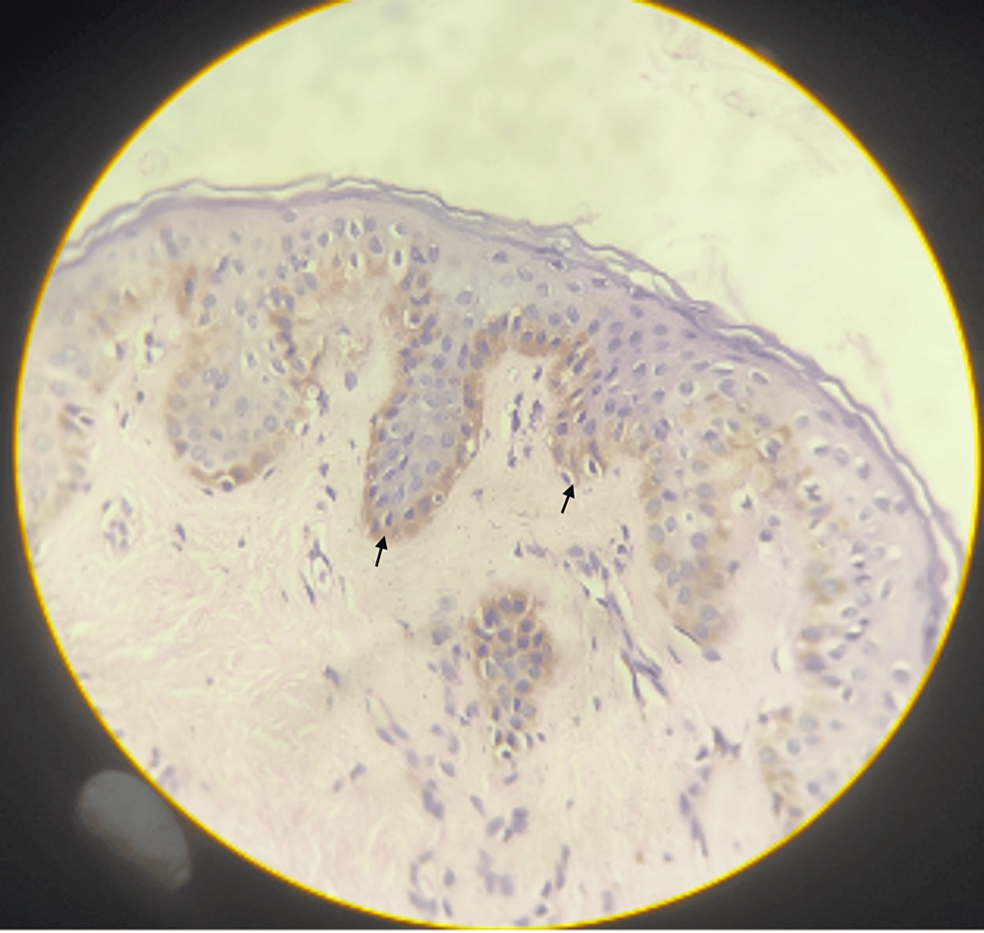
Epidermis showing hyperkeratosis, parakeratosis, irregular acanthosis, and spongiosis with focal ballooning of keratinocytes.

**Figure 6:**
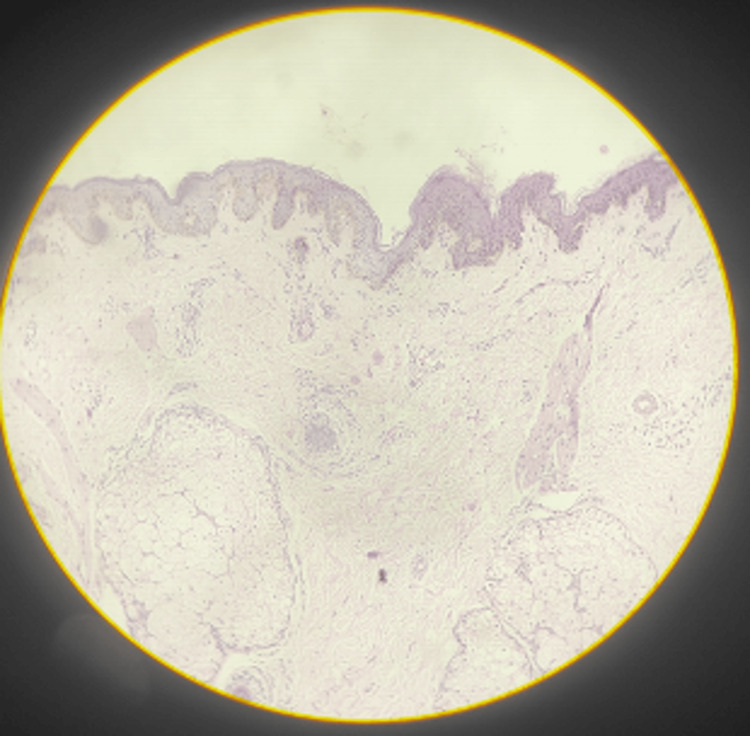
Dermis showing edema with perivascular lymphocytic and eosinophilic infiltrate.

On histopathological examination, the epidermis showed hyperkeratosis, parakeratosis, irregular acanthosis (indicated by the black arrow), and spongiosis with focal ballooning of keratinocytes in the upper dermis (Figure 5). The underlying dermis shows edema with perivascular lymphocytic and eosinophilic infiltrate (Figure 6).

**Figure 7:**
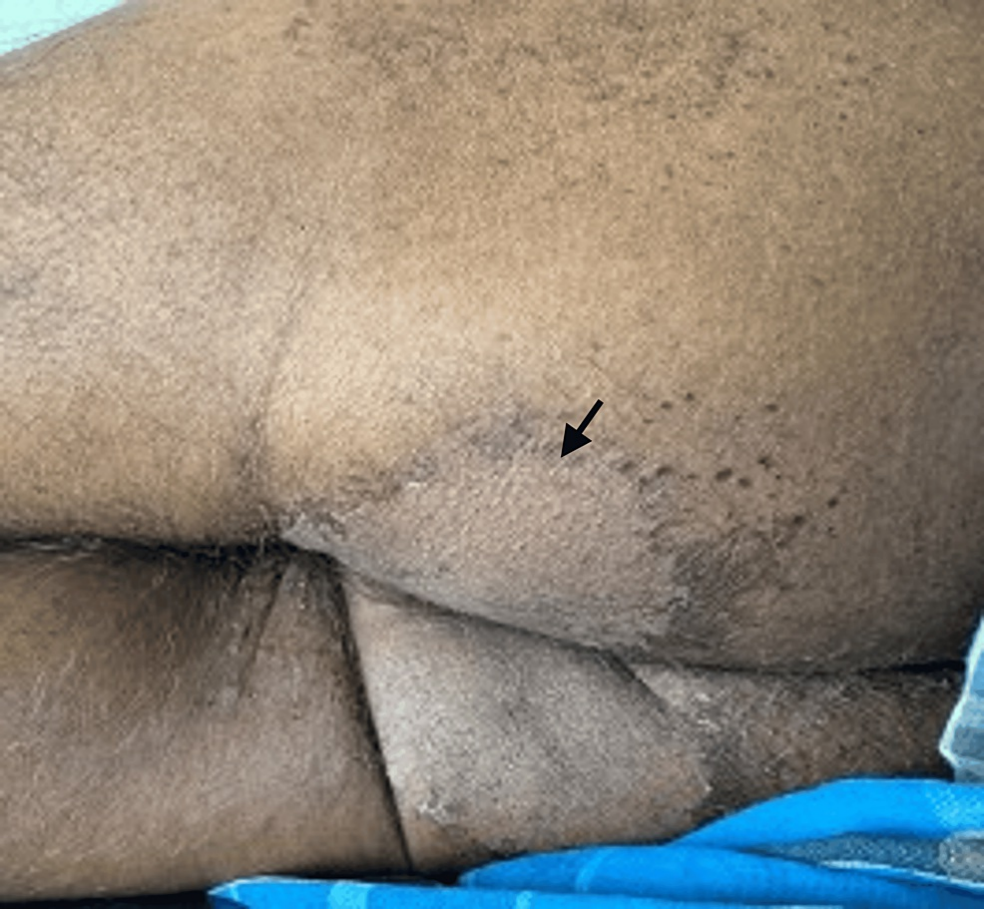
Improvement of skin lesions over the gluteal region following nutritional supplementation.

**Figure 8:**
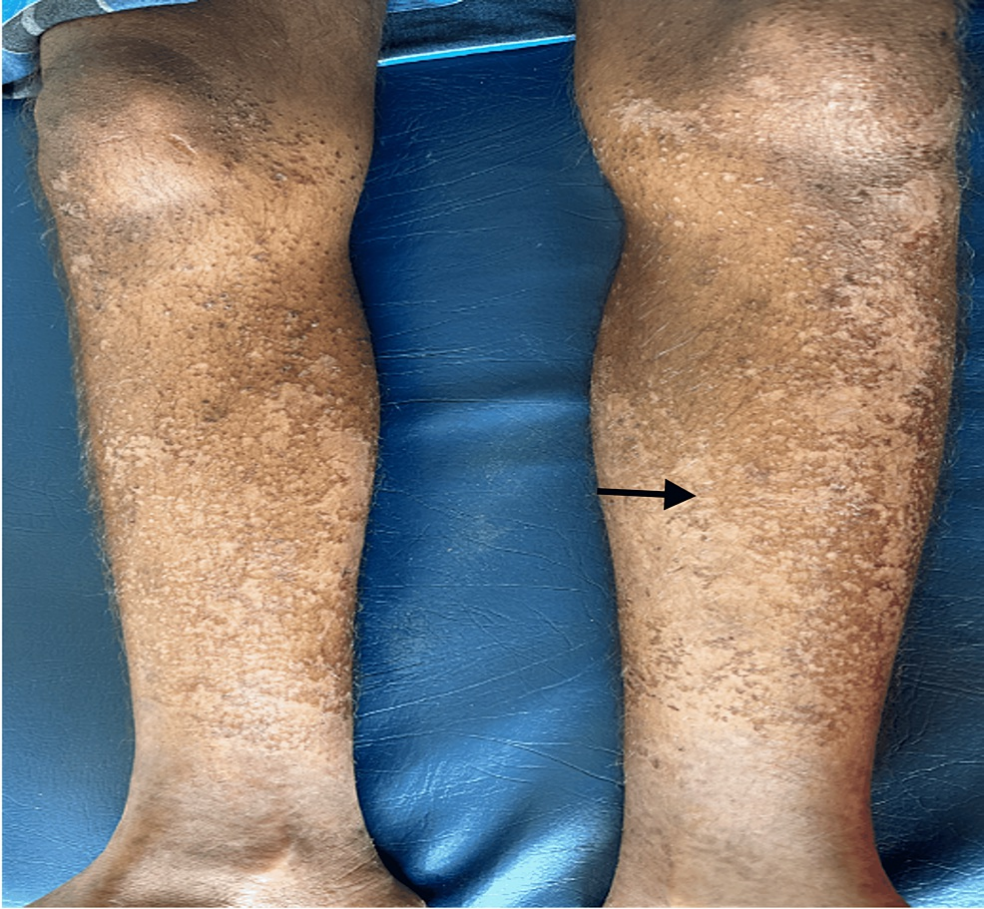
Improvement of skin lesions over the lower limbs following nutritional supplementation.

Based on clinical and histopathological findings, a diagnosis of pseudoglucagonoma syndrome was made. The patient was treated with topical corticosteroids, emollients, oral vitamin D, methylcobalamin, vitamin B complex, folic acid, alpha lipoic acid, inositol, amino acids, zinc, and iron supplements. Improvement in skin lesions was observed with improved nutrition (Figures 7-8).

## Discussion

Pseudoglucagonoma syndrome is defined as the presence of NME in the absence of a glucagon-secreting tumor [1]. It is extremely rare [1], and the diagnosis is often delayed, resulting in delayed treatment [2]. It may occur secondary to pancreatitis, pancreatic insufficiency, gastrointestinal dysfunction, inflammatory bowel disease, celiac disease, malabsorption disorders, nutritional deficiencies, hepatocellular dysfunction, and hypoalbuminemia [2].

Glucagonoma syndrome caused by glucagon-secreting pancreatic tumor was first described by Becker et al. in 1942 [3]. NME, the hallmark of this syndrome, is characterized by pruritic or painful erythematous well-demarcated plaques or patches with irregular edges, predominantly distributed in areas of increased friction and pressure [4]. They spread in a centrifugal pattern and may form flaccid bullae that erode and crust, eventually healing with hyperpigmentation [4]. These lesions are cyclical, typically waxing and waning for about 10 days, and are in different stages of healing [4]. The histopathological features of NME include parakeratosis, irregular acanthosis with loss of the granular layer, ballooning of epidermal cells showing vacuolar degeneration, particularly in the upper spinous layer, intraepidermal bulla, and mild perivascular lymphocytic or neutrophilic infiltrate [4]. The hallmark of NME is epidermal necrosis [5]. The differential diagnosis of NME includes acrodermatitis enteropathica, essential fatty acid deficiency, annular lupus erythematosus, drug reactions, contact dermatitis, and deficiency of vitamins such as niacin, riboflavin, or pyridoxine [6].

The exact cause is unknown [6]. NME can be classified as a deficiency dermatosis [6]. The multifactorial malnutrition model of NME states that hyperglucagonemia induces a catabolic state resulting in hypoaminoacidemia, deficiency of zinc and essential fatty acids, and the induction of inflammatory mediators in the epidermis contributing to the epidermal dysfunction [6]. Pseudoglucagonoma syndrome occurs in the absence of hyperglucagonemia [6]. It is mediated by enteroglucagon, which is produced by the crypt cells of the pancreas in the malabsorptive state [6].

NME in the absence of glucagonoma has been reported in a heroin-dependent patient [7], iatrogenic case [8], associated with zinc deficiency [9], and secondary to acute pancreatitis who were alcohol-dependent [10]. Pseudoglucagonoma syndrome has also been reported in a malnourished patient after a large resection of the colon and pancreatojejunostomy [10].

Hariri et al. [2] reported a case of pseudoglucagonoma syndrome who presented with erythematous patches with hyperpigmented scaly borders over the palms and soles. Improvement of the lesions was observed with improved nutrition.

Kumari et al. [1] reported a case of pseudoglucagonoma syndrome secondary to chronic pancreatitis. Improvement of skin lesions was observed with emollients, topical steroids, intravenous protein infusions, and other supplements.

## Conclusions

NME associated with glucagonomas or as a part of pseudoglucagonoma syndrome runs a variable course and tends to have a good prognosis, with rapid resolution of skin lesions following surgical resection of the tumor or nutritional correction. A high degree of suspicion is needed when erythematous necrolytic skin lesions resembling NME may occur in malnourished patients who have undergone major gut surgeries, and appropriate investigations to rule out internal associations could help in effective management. This case of NME in a patient with pseudoglucagonoma following Frey’s procedure is being reported for its rarity. A literature search has revealed only five case reports on pseudoglucagonoma syndrome. To the best of our knowledge, this is the first case report of pseudoglucagonoma syndrome following Frey’s surgery.

## References

[1] Kumari YA, Reddy IC, Sree KG, Ala M (2015). Pseudoglucagonoma syndrome secondary to pancreatitis: a case report. J NTR Univ Health Sci.

[2] Hariri JO, Abduljabbar MH, Alshammari SKM (2020). Pseudogluoconoma: a case report. IJMDC.

[3] Dal Coleto CC, de Mello APF, Piquero-Casals J, Lima FR, Vilela APC, Festa-Neto C, Sanches JA (2001). Necrolytic migratory erythema associated with glucagonoma syndrome: a case report. Rev Hosp Clín Fac Med S Paulo.

[4] Foss MG, Hashmi MF, Ferrer-Bruker SJ (2023). Necrolytic Migratory Erythema. https://www.ncbi.nlm.nih.gov/books/NBK532872/.

[5] Teixeira RC, Nico MM, Ghideti AC (2008). Necrolytic migratory erythema associated with glucagonoma: a report of 2 cases. Clinics (Sao Paulo).

[6] https://www.medscape.com/viewarticle/487806_4.

[7] Sinclair SA, Reynolds NJ (1997). Necrolytic migratory erythema and zinc deficiency. Br J Dermatol.

[8] Echenique-Elizondo M, Valls AT, Orue JLE, de Lizarduy IM, Aguirre JI (2004). Glucagonoma and pseudoglucagonoma syndrome. JOP.

[9] Echenique-Elizondo M, Elorza JL, De Delas JS (2003). Migratory necrolytic erythema and glucagonoma. Surgery.

[10] Bak H, Ahn SK (2005). Pseudoglucagonoma syndrome in a patient with malnutrition. Arch Dermatol.

